# Bilayer Effects of Antimalarial Compounds

**DOI:** 10.1371/journal.pone.0142401

**Published:** 2015-11-09

**Authors:** Nicole B. Ramsey, Olaf S. Andersen

**Affiliations:** 1 Weill Cornell/Rockefeller/Sloan-Kettering Tri-Institutional MD-PhD Program, New York, NY, United States of America; 2 Graduate Program in Pharmacology, Weill Cornell Graduate School of Medical Sciences, New York, NY, United States of America; 3 Department of Physiology and Biophysics, Weill Cornell Medical College, New York, NY, United States of America; University of Michigan, UNITED STATES

## Abstract

Because of the perpetual development of resistance to current therapies for malaria, the Medicines for Malaria Venture developed the Malaria Box to facilitate the drug development process. We tested the 80 most potent compounds from the box for bilayer-mediated effects on membrane protein conformational changes (a measure of likely toxicity) in a gramicidin-based stopped flow fluorescence assay. Among the Malaria Box compounds tested, four compounds altered membrane properties (p< 0.05); MMV007384 stood out as a potent bilayer-perturbing compound that is toxic in many cell-based assays, suggesting that testing for membrane perturbation could help identify toxic compounds. In any case, MMV007384 should be approached with caution, if at all.

## Introduction

Malaria continues to be a dangerous illness, causing 584,000 fatalities in impoverished, tropical regions in 2010 [1]. The pathogenesis of the disease and the perpetual emergence of resistance necessitates combination therapy and the need for continuous addition of new drugs to the pipeline [2]. Yet, despite continued efforts to develop new drugs, there currently are no approved drugs for malaria that do not have documented resistance [3].

This is a serious problem because, in the case of neglected tropical diseases (NTDs) that predominantly infect people in developing nations, the cost of developing a drug is a critical limiting factor. It thus becomes important to develop tools to identify likely failures early in development, and thereby decrease the cost of drug discovery and development. A key concern in this context is safety, given the populations that are most at risk of succumbing to malaria and the need for widespread administration in an asymptomatic population, as recommended by the current eradication agenda [4].

To facilitate the development of novel drug candidates and biological probes for treating and studying malaria, the Medicines for Malaria Venture (MMV, http://www.mmv.org) began distributing the Malaria Box in 2012 [5]. The compounds chosen for inclusion in the Malaria Box were selected based on collective phenotypic screening at GlaxoSmithKline, Novartis, and St. Jude Children’s Research Hospital. 30,000 unique structures displayed phenotypic hits at submicromolar concentrations, and the MMV included a limited diverse set that had undergone principal component analysis, physicochemical profiling, and determination of commercial availability. This final product was split into two categories of drug-like and probe-like using Rule-of-Five compliance [6] and substructure filters to eliminate molecules that for a variety of reasons are likely to be problematic [7] including PAINS (pan assay interference compounds) [8].

Due to the predominantly intracellular nature of the malaria life cycle, successful drugs will need to cross cell membranes—and, unless the compound can enter cells by a protein-catalyzed process [9], it will need to traverse the membrane’s lipid bilayer [10]. Such uncatalyzed solute movement across lipid bilayers involves partitioning into the bilayer and diffusion through the hydrophobic core (the solubility-diffusion mechanism [11]. The joint requirements, that successful antimalarials need to be both sufficiently water-soluble to distribute throughout the body and sufficiently lipid-soluble to cross cell membranes to reach their intracellular targets, entail that antimalarials, like many other drugs, are amphiphiles–as would be expected from Lipinski’s Rule of Five [6, 12].

It long has been appreciated that drugs may alter lipid bilayer properties [13], and drug-induced in lipid bilayer structure and dynamics have been demonstrated using a variety of spectroscopic methods in the case of antidepressants [14, 15] phytochemicals [16, 17] and antimicrobial peptides [18, 19]—and many biologically active amphiphiles alter lipid bilayer properties and integral membrane protein function at similar concentrations [20–25]. This bilayer regulation of membrane protein function arises because membrane protein function depends on conformational transitions that involve the proteins’ bilayer-spanning domains–leading to alterations in lipid packing adjacent to the proteins of interest. Such bilayer deformations incur an energetic cost, the bilayer deformation energy (Δ*G*_def_) such that the free energy difference between different protein conformations (I and II) includes a contribution from the difference in bilayer deformation energy associated with the two protein conformations: $\Delta G_{\text{total}}^{\text{I}\to \text{II}}=\Delta G_{\text{protein}}^{\text{I}\to \text{II}}+\Delta G_{\text{bilayer}}^{\text{I}\to \text{II}}$, where $\Delta G_{\text{protein}}^{\text{I}\to \text{II}}$ denotes the energetic contribution from rearrangements within the protein and $\Delta G_{\text{bilayer}}^{\text{I}\to \text{II}}(=\Delta G_{\text{def}}^{\text{II}}-\Delta G_{\text{def}}^{\text{I}})$ the contribution from the bilayer (the different lipid packing associated with the different conformations).

$\Delta G_{\text{def}}^{\text{I}}$ and $\Delta G_{\text{def}}^{\text{II}}$, and thus $\Delta G_{\text{bilayer}}^{\text{I}\to \text{II}}$, vary as function of the bilayer physical properties [26, 27], meaning that changes in bilayer lipid composition, such as the addition or removal of cholesterol or the partitioning of amphiphiles into the lipid bilayer/solution interface (and the ensuing changes in bilayer properties), will alter membrane protein function. Experimental maneuvers that increase bilayer stiffness, such the addition of cholesterol, thus will alter membrane protein function [28], with the direction of the change determined by the changes in $\Delta G_{\text{def}}^{\text{II}}$ relative to the changes in $\Delta G_{\text{def}}^{\text{I}}$. Amphiphile-induced changes in lipid bilayer properties similarly have been observed with different classes of molecules: detergents and lipid metabolites including poly-unsaturated fatty acids [22, 29–31], phytochemicals [16, 20, 23, 25], and drugs [14, 24], see also [32].

Membrane effects have been correlated with toxicity and screening for membrane effects have been proposed as a cost and time effective way to measure toxicity, using lipid vesicle-based systems [33], and drugs that are potent modifiers of membrane (lipid bilayer) properties previously have been shown to have an increased risk of failing as safe drugs [24]. We therefore decided to explore whether antimalarial drug candidates cause membrane effects, and, if so, whether these effects are large enough to be of concern—whether the drugs are likely to produce indiscriminate changes in membrane protein function that would be expected to cause undesired effects.

Screening drug candidates for bilayer effects thus might reduce the resources spent on drug development by providing information about small molecule promiscuity at an early stage in development. Using the eighty most potent Malaria Box compounds (40 drug-like and 40-probe-like), we screened for membrane (off-target) effects using a gramicidin-based assay that has been validated to predict membrane-mediated effects on integral membrane proteins [21, 24, 25, 34, 35].

Gramicidin channels form by transmembrane dimerization of non-conducting monomers, and channel formation leads to a local bilayer thinning with an energetic cost that varies with (drug-induced) changes in lipid bilayer properties. Changes in the equilibrium distribution between non-conducting monomers and conducting dimers can be quantified using electrophysiological [32] and fluorescence [36] methods. We used a gramicidin-based fluorescence assay (GBFA) that employs fluorophore-loaded large unilamellar vesicles (LUVs) doped with gramicidin and a gramicidin channel-permeable heavy ion quencher (Tl^+^), where changes in the gramicidin monomer↔dimer equilibrium are monitored as changes in the time course of fluorescence quenching. The assay has previously been used to screen a library of compounds that inhibited acid-base homeostasis in *M*. *tb*. [37], where over half of the candidate hits identified in a whole screen (for inhibitors of pH homeostasis) altered bilayer properties. Would the Malaria Box compounds have a similar proportion of compounds that modulate bilayer properties?

## Materials and Methods

LUVs were prepared from 1,2-dierucoyl-*sn*-glycero-3-phosphocholine in chloroform (Avanti Polar Lipids, Alabaster, AL) and gramicidin (Sigma Chemical Co, St. Louis, MO) in methanol (lipid:gramicidin molar ratio 2000:1), and loaded with the fluorophore 8-aminonaphthalene-1,3,6-trisulfonic acid (ANTS) disodium salt (Invitrogen, Eugene, OR) using a freeze-thaw and extrusion method, as described previously [36, 38, 39]. The lipids and gramicidin were dissolved in chloroform:methanol, the solvents were removed by dried under nitrogen until the lipid:gramicidin mixture appeared to be dry and followed by incubation in a desiccator overnight. The dried lipid:gramicidin film was rehydrated in 100 mM NaNO_3_, 25 mM ANTS, and 10 mM HEPES (pH 7.0) at room temperature for 3–4 hours or overnight. The mixture was sonicated at low power for 1 min, subjected to 5 freeze–thaw cycles, and extruded 21 times, at room temperature, using an Avanti mini-extruder or Lipex 10 mL extruder and a 0.1 μm polycarbonate membrane filter. Extravesicular ANTS was removed using a PD-10 Desalting column (GE Healthcare, Piscataway, NJ), and the ANTS-LUV stock solution was stored at 12°C, in the dark, for a maximum of 10 days. The ANTS-LUV stock solution was diluted 1:20 with 140 mM NaNO_3_ plus 10 mM HEPES (pH 7.0) after incubating for 24 h at 12°C in the dark.

All molecules were provided by the MMV in the 80 compound Malaria Box; they were diluted in DMSO and stored at -40°C.

The GBFA was performed as described previously [39]. The time course of fluorescence quenching was measured at 25°C using an SX.20 Stopped-Flow Spectrometer (Applied Photophysics, Leatherhead, UK), with a dead time ∼1.2 ms. The excitation was at 352 nm and the fluorescence was recorded above 455 nm using a high-pass filter and a sampling rate of 5,000 points/s. For each experimental condition, at least 5 repeated mixing trials were measured, from 1 or more vesicle preparations. Because of the inevitable variation in LUV sizes, the time course of fluorescence quenching was normalized to the average fluorescence without quencher for that sample, and the first 2–100 ms was fitted with a stretched exponential [36, 40]. The initial influx rate was estimated from the rate of the stretched exponential at 2 ms and normalized to the influx rate in the presence of vehicle only. Unpaired t-tests were performed using PRISM (GraphPad Software, Inc., La Jolla, CA). *P* values less than 0.05 were considered statistically significant.

## Results

**Fig 1:**
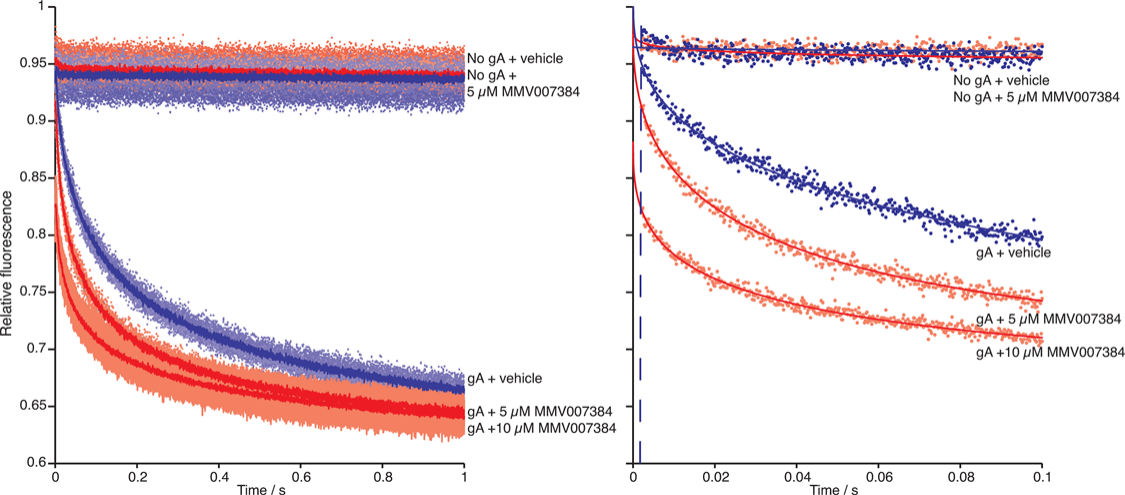
MMV007384 is a bilayer-modifying compound. Fluorescence quench traces showing the effects of 5,5'-Methylenebis [2- (4-methoxyphenyl)-1H-benzimidazole] or MMV007384 [5]) in the gramicidin-based fluorescence assay (GBFA). The panel left shows the results over 1 s. In the presence of 5 or 10 μM of the Malaria Box compound, the quench rate normalized to control (in the absence of the compound) increased to 1.8 (q_5_) and 2 (q_10_), respectively. The difference is statistically significant (p < .0001). The right panel shows the average trace for a single run at higher time resolution.

**Fig 2:**
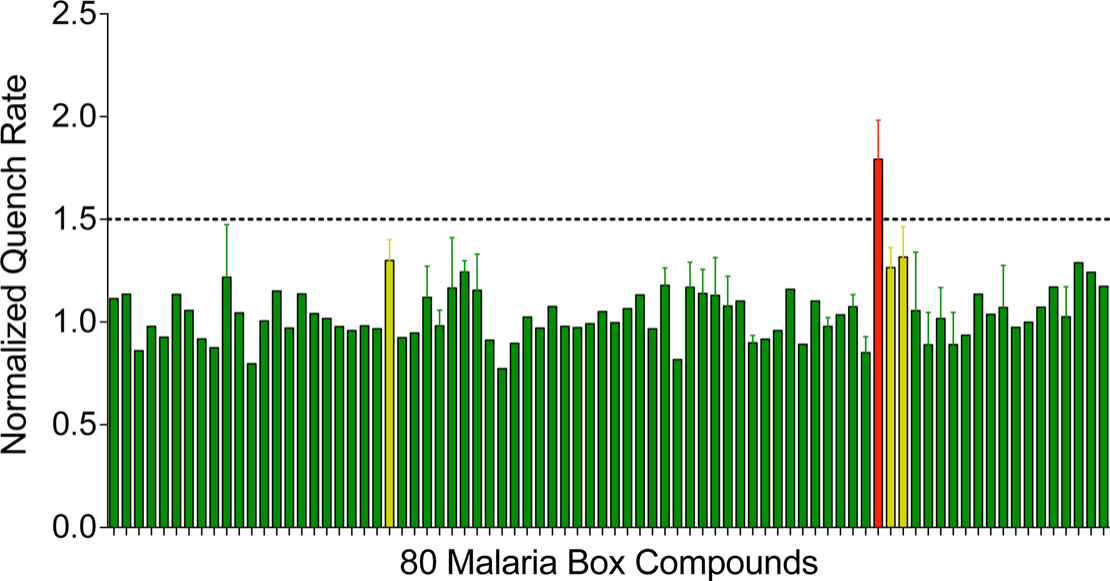
Four Malaria Box compounds have statistically significant bilayer effects. 80 Malaria Box compounds were tested at 5 μM, the order of the compounds is A2 … A11, B2 … B11, etc. The four Malaria Box compounds that perturb the bilayer are MMV007384 (red bar, q_5_ = 1.79, p<0.01), and from L to R (amber bars): N-{4-[(4-methyl-1-piperidinyl)methyl]phenyl}-1H-pyrrolo[3,2-h]quinoline-2-carboxamide, MMV020549, q_5_ = 1.3, p<0.05); N-{4-[(4-ethyl-1-piperazinyl)methyl]phenyl}-1H-pyrrolo[3,2-h]quinoline-2-carboxamide, MMV020548, q_5_ = 1.27, p < .05); and 2-{[1-(2,4-dimethoxyphenyl)-1H-imidazol-2-yl]sulfanyl}-N-(2,5-dimethylphenyl)acetamide MMV019258, q_5_ = 1.32, p < .05). Error bars depict S.E.M. Unpaired, two-tailed t-tests were performed in comparison to control.

Fig 1 shows results obtained when fluorophore-loaded, gramicidin-doped LUVs are mixed with a gramicidin channel-permeant quencher (Tl^+^) in a stopped-flow spectrofluorometer. The fluorescence decrease reflects the influx of Tl^+^ into the vesicle, which quenches the fluorophore (ANTS) fluorescence. In the absence of gramicidin, there is no change in fluorescence. The fluorescence quench rate was accelerated in the presence of a bilayer-modifying amphiphile, such as MMV007384, which is 5,5'-Methylenebis [2- (4-methoxyphenyl)-1H-benzimidazole] [5]. Using quench rates determined from the fluorescence traces (similar to those depicted in Fig 1), dose-response curves (1–10 μM) were constructed for each compound; the results are summarized in S1 Fig. To visualize whether the Malaria Box compounds alter lipid bilayer properties, Fig 2 shows the normalized rates (relative to control, in the absence of the compound in question) for each compound at 5 μM (the Malaria Box compounds’ EC_50_ vary between ∼10 nM and 3 μM [5], and MMV recommends testing at 1 μM; we chose the results obtained at 5 μM to visualize whether or not the compounds altered the fluorescence quench rate). Four of the 80 Malaria Box compounds alter lipid bilayer properties as evident by increased quench rates as compared to control (the chemical structures of these four compounds are depicted in S2 Fig).

Whether or not the Malaria Box compounds altered lipid bilayer properties, they did not compromise the lipid bilayer barrier properties—there was no evidence for leakage of ANTS out of the LUVs during the 10 min incubation with 5 or 10 μM of the Malaria Box compounds (which would be observed as an instantaneous decrease in fluorescence when the LUVs were mixed with the Tl^+^-containing quench solution). Nor did the Malaria Box compounds (other than the four in Fig 2) increase the rate of Tl^+^ influx, in the absence or presence of gramicidin, meaning that they did not produce leaks in the LUV membrane.

The most potent of the four membrane-active Malaria Box compounds, MMV007384, is classified by MMV as a probe-like compound and has a bilayer-modifying potency that is correlated with toxicity to cells in culture [41]. In fact, MMV007384 is known to have a high IFI Promiscuity Index (S3 Fig), which is a measure of activity in high-throughput toxicity assays performed at Novartis-GNF or GSK, as well as low IC_50_s for drug-resistant strains of *P*. *falciparum* and several NTDs such as *T*. *brucei rhodesiense*, *L*. *infantum*, *T*. *brucei*. *T*. *cruzi*, *M*. *tuberculosis* and cytotoxicity against MRC-5 human fibroblasts and the human hepatoma cell line Huh7 (S4 Fig).

**Fig 3:**
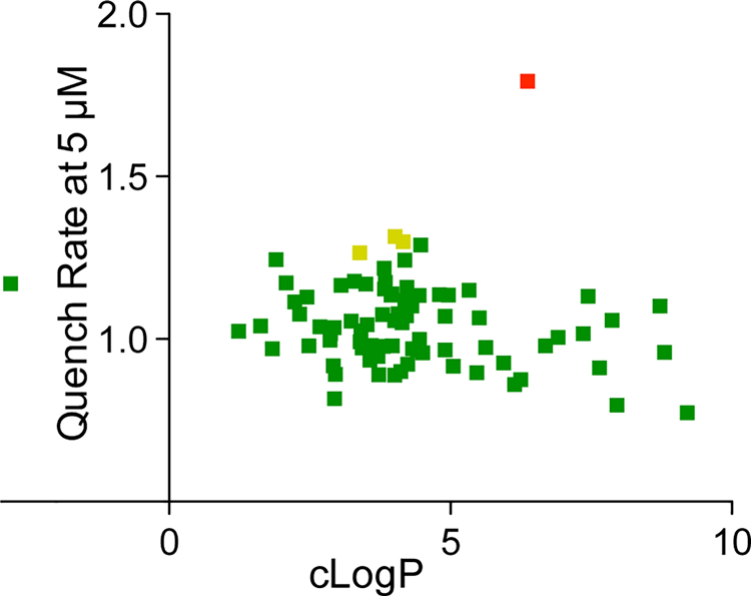
The Malaria Box compounds’ estimated hydrophobicity (clogP) is not correlated with lipid bilayer effects. Plot of the quench rate (relative to control) vs. cLogP, which was calculated using the ALOGP algorithm [44] as implemented by Collaborative Drug Discovery, Inc. (http://collaborativedrug.com, Burlingame, CA). MMV007384 (red dot cLogP = 6.37); from L to R (amber dots): MMV020548, cLogP = 3.39; MMV020549, cLogP = 4.16; and MMV019258, cLogP = 4.01.

**Fig 4:**
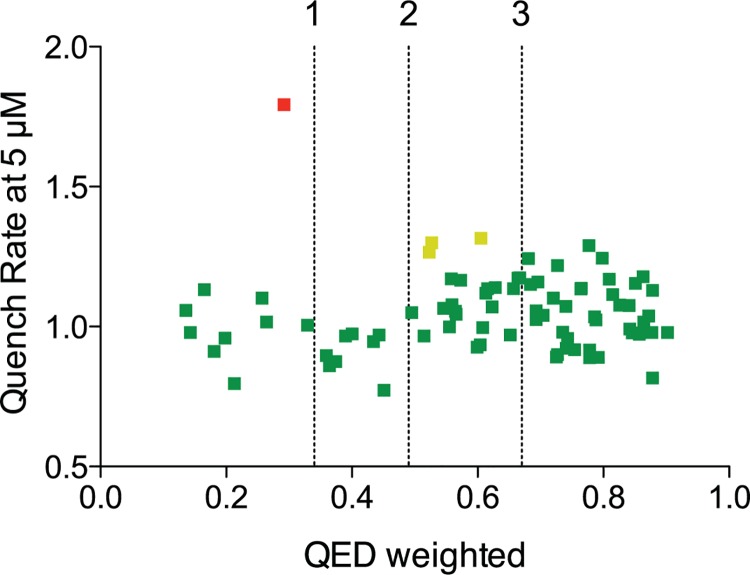
QED does not correlate with bilayer-mediated off-target effects. Plot of the quench rate (relative to control) vs. the weighted QED [43]. There is some clustering of the Malaria Box compounds toward the “attractive” QED value. MMV007384 (red dot, weighted QED = .292) is an unattractive complex compound. **From L to R** (amber dots): MMV020548 (weighted QED = .523) and MMV020548 (weighted QED = .527) are unattractive complex compounds; and MMV019258 (weighted QED = .605) is at the cusp of attractive and unattractive simple compounds. Vertical dotted lines represent reported averages of, from L to R: unattractive (complex); unattractive (simple); and attractive QED values. QED was calculated using Pipeline Pilot (Accelrys Inc., San Diego, CA). (Weighted QED did not differ substantially from unweighted QED, not shown).

Many drug-screening assays use physicochemical parameters and substructure filters, e.g. calculated octanol/water partition coefficients (cLogP) [42], Lipinski’s “Rule of Five” [6], or the Rapid Elimination of Swill (REOS) [7], the screen for Pan Assay Interference Compounds (PAINS) [8], or the Quantitative Estimate of Drug-likeness (QED) [43], to evaluate/predict a compound’s drug-likeness, bioavailability or toxicity. We analyzed the Malaria Box compounds according to physicochemical parameters like cLogP (Fig 3), Lipinski’s Rule of Five (five of the 80 Malaria Box compounds violated two of Lipinski’s guidelines) and QED (Fig 4, S5 Fig), as calculated using Pipeline Pilot (Accelrys Inc., San Diego, CA).

The Malaria Box compounds’ cLogP values are distributed between -2.5 and 10, with the drug-like compounds having cLogP values between 1 and 5 (in accordance with Lipinski’s Rule of Five; by design, most Malaria Box drug-like compounds fail none of the criteria in the Rule of Five). When evaluating the Malaria Box compounds using the quantitative estimate of drug-likeness (QED) measure [43] there is clustering around “attractive” QED (high drug-likeness) values (Fig 4). Overall, there is little correlation between bilayer-perturbing effects and any of the “standard” measures of drug-likeness, suggesting that the GBFA is a novel tool to be considered when evaluating the likely toxicity of drug candidates. Employing the GBFA is concordant with the “quick win, fast fail” drug development paradigm, which proposes that technical uncertainty be decreased before expensive later development stages are embarked upon [45].

## Discussion

In this study, we explored whether the compounds provided in the Malaria Box have significant bilayer-modifying effects. We find that, as might have been predicted *a priori* based on the criteria used to select the Malaria Box compounds, that only 5% of the compounds have statistically significant effects on lipid bilayer properties. Only one of these, MMV007384, alters the fluorescence quench more than 50%, suggesting that it might alter membrane protein function and thereby cause unwanted effects. Indeed, MMV007384 is categorized as a probe-like compound with considerable toxicity in cellular studies (https://www.ebi.ac.uk/chembl/), suggesting that studies on the bilayer-modifying effect of drug candidates may provide additional information on their safety (as well as identifying possible mechanisms for any toxicity).

It is in this context important that antimalarials need to cross at least one cell membrane in order to exert their action. They can do so via protein-catalyzed transfer [9, 10] or by the solubility-diffusion mechanism [11]; in the latter case, the drugs will partition into the lipid bilayer/solution interface and thereby alter lipid bilayer properties. The question thus becomes whether the changes in bilayer properties are of sufficient magnitude to be relevant because different classes of drugs have different bilayer-perturbing propensities, e.g., [24, 35]. A previous screen for compounds that disrupt the pH homeostasis in *M*. *tb*. [37], as well as a recent study on phytochemicals [25] shows that many biologically active compounds have the capacity to be bilayer-perturbing at the concentrations where they alter membrane protein function, and membrane-active compounds such as capsaicin and curcumin alters lipid bilayer structure and dynamics as visualized using nuclear magnetic resonance [16, 17]. These results, together with previous studies on the bilayer-perturbing effects of biologically active compounds [20–22, 24, 25, 31, 46], further demonstrate that bilayer-modifying effects are generalizable, in the sense that the changes in gramicidin channel function are similar in bilayers of different composition [20, 22, 24, 31, 46], including cell membranes [47]—and that one can predict amphiphile-induced changes in integral membrane protein function from the changes in gramicidin channel function in the synthetic systems we have developed [21, 24, 25].

The release of the Malaria Box has stimulated studies on the compounds provided in the box, leading to a number of discoveries. In studies on *P*. *falciparum*: Fletcher and Avery [48] showed that 12 Malaria Box compounds are potential inhibitors of the Coenzyme A pathway; Hain et al. [49] identified a target (Atg8-Atg3 protein-protein interaction) for six Malaria Box compounds in the parasite’s blood and liver stages; Lucantoni et al. [50] found 64 gametocidal compounds amongst the Malaria Box compounds; and Paiardini et al. [51] found two compounds active against neutral aminopeptidases M1 and M17. In studies on other parasites: Boyom et al. [52] showed that seven Malaria Box compounds are potent inhibitors of *Toxoplasma gondii*, and two are potent inhibitors of *Entamoeba histolytica*; Alemán Resto et al. [53] identified three compounds that may target the oyster Dermo Disease caused by *Perkinsus marinus*; Njuguna et al. [54] identified two compounds that appear to target the tick *Theileria parva*, which causes East Coast Fever in cattle; and Kaiser et al. [55] found nine promising *in vitro* candidates that inhibited growth of *Trypanosoma* and *Leishmania* species.

Three of the compounds identified in the above studies (MMV007384, MMV020549 and MMV020548) are among those we have shown to perturb the lipid bilayer. Compounds MMV007384 and MMV020548 are potent gametocidal agents [50], and MMV020549 has activity against *E*. *histolytica* [52]. These compounds should be approached with caution given the likelihood of membrane effects, which is likely to produce undesired toxicity in long-term therapy (or prophylaxis among asymptomatic patients), a concern that is magnified by to the need to use concentrations that are ten times higher than EC_50_ in order to inhibit a target by 90%.

Given the results on compounds that disrupt pH homeostasis in *M*. *tb*. [37], it is surprising that only four of the compounds had a significant effect on membrane properties. We attribute the difference between the results in the current study and those of Darby et al. to the different stages where the two groups of compounds are in the discovery process. Compared to the tuberculosis compounds, the proportion of Malaria Box compounds that are membrane active is much less, most likely because the stage of the discovery process for the Malaria Box compounds is more advanced. We are pleased to report that 76 of the 80 most potent Malaria Box compounds have no discernible bilayer effects at concentrations up to 10 μM, meaning that they are unlikely to have side effects associated therewith.

## Supporting Information

S1 Fig Dose-response curves for the Malaria Box compounds’ bilayer-modifying propensities using the GBFA. Eighty molecules were tested; the results are separated into eight graphs, each depicting results for 10 molecules, which are identified by their well positions in the Malaria Box plate. The interrupted lines at 1.5 on the y-axes correspond a cut-off for bilayer-mediated effects when interpreting the GBFA results. The compounds identities at the indicated plate positions can be found in Table S1 from Spangenberg et al. [5]. (MMV020549 is C4; MMV007384 is G3; MMV020548 is G4; and MMV019258 is G5).
(EPS)

S2 Fig Chemical Structures of the bilayer-perturbing Malaria Box molecules. These four molecules, at 5 μM, increase the fluorescence quench rates statistically significantly in comparison to control (p<0.05; p<0.01 for MMV007384).
(TIFF)

S3 Fig MMV007384 is toxic in many assays of cell toxicity. MMV007384 is highly active in toxicity assays done by GNF-Novartis. The figure depicts the fluorescence quench rates as a function of each compound’s IFI Promiscuity Index (the Inhibition Frequency Index is the fraction of the HTS screens where a compound has been used and showed more than 50% inhibition). MMV007384 (marked in red) stands out by having the highest IFI Promiscuity Index (>0.25) and the largest increase in quench rate. IFI Promiscuity Indices for the different molecules were from Collaborative Drug Discovery, Inc. (http://collaborativedrug.com Burlingame, CA 94010).
(EPS)

S4 Fig MMV007384 is highly toxic to HuH7 cells. Each molecule’s cytotoxicity was quantified by its CC_50_ (the concentration, in μM, required to inhibit 50% *of cell growth*); MMV007384 is marked in red. Most of the Malaria Box compounds have CC_50_’s on HuH7 cells (a human hepatocellular carcinoma line http://huh7.com/) less than 0.15. Only compounds that were tested by Novartis-GNF are plotted in this graph. CC_50_ values were from Collaborative Drug Discovery, Inc. (http://collaborativedrug.com, Burlingame, CA 94010).
(EPS)

S5 Fig Malaria Box compounds’ QED scores. The Malaria Box compounds tend to have “attractive” QED scores (mean = 0.619, median = 0.666), with no apparent difference between weighted and unweighted scores (not shown). Vertical dotted lines represent reported averaged of, from L to R—unattractive (complex), unattractive (simple), and attractive QED values. The red arrow marks MMV007384. Weighted QED did not differ substantially from unweighted QED (not shown). QED values were calculated using Pipeline Pilot (Accelrys, Inc., San Diego, CA) following [43].
(EPS)

## References

[1] World Health Organization. WHO World Malaria Report 2011. Geneva, Switzerland (http://www.who.int/malaria/world_malaria_report_2011/en/).

[2] Dandapani S, Comer E, Duvall JR, Munoz B. Hits, leads and drugs against malaria through diversity-oriented synthesis. Future Med Chem. 2012;4(18):2279–94. 10.4155/fmc.12.178 . 23234551

[3] Phyo AP, Nkhoma S, Stepniewska K, Ashley EA, Nair S, McGready R, et al Emergence of artemisinin-resistant malaria on the western border of Thailand: a longitudinal study. Lancet. 2012;379(9830):1960–6. 10.1016/S0140-6736(12)60484-X 22484134 PMC3525980

[4] The malERA Consultative Group on Drugs. A research agenda for malaria eradication: drugs. PLoS Med. 2011;8(1):e1000402 10.1371/journal.pmed.1000402 21311580 PMC3026688

[5] Spangenberg T, Burrows JN, Kowalczyk P, McDonald S, Wells TN, Willis P. The open access Malaria Box: a drug discovery catalyst for neglected diseases. PloS one. 2013;8(6):e62906 10.1371/journal.pone.0062906 23798988 PMC3684613

[6] Lipinski CA, Lombardo F, Dominy BW, Feeney PJ. Experimental and computational approaches to estimate solubility and permeability in drug discovery and development settings. Adv Drug Deliv Rev. 1997;23:3–26. 10.1016/s0169-409x(00)00129-0 11259830

[7] Walters WP, Namchuk M. Designing screens: how to make your hits a hit. Nat Rev Drug Discov. 2003;2(4):259–66. 10.1038/nrd1063 . 12669025

[8] Baell JB, Holloway GA. New substructure filters for removal of pan assay interference compounds (PAINS) from screening libraries and for their exclusion in bioassays. J Med Chem. 2010;53(7):2719–40. 10.1021/jm901137j . 20131845

[9] Desai SA. Ion and nutrient uptake by malaria parasite-infected erythrocytes. Cellular microbiology. 2012;14(7):1003–9. 10.1111/j.1462-5822.2012.01790.x 22432505 PMC3381363

[10] Basore K, Cheng Y, Kushwaha AK, Nguyen ST, Desai SA. How do antimalarial drugs reach their intracellular targets? Frontiers in pharmacology. 2015;6:91 10.3389/fphar.2015.00091 25999857 PMC4419668

[11] Finkelstein A. Water Movement Through Lipid Bilayers, Pores, and Plasma Membranes. Theory and Reality. New York: John Wiley; 1987.

[12] Lundbæk JA. Lipid bilayer-mediated regulation of ion channel function by amphiphilic drugs. J Gen Physiol. 2008;131(5):421–9. 10.1085/jgp.200709948 18411332 PMC2346573

[13] Seeman P. The membrane actions of anesthetics and tranquilizers. Pharmacol Rev. 1972;24(4):583–655. 4565956

[14] Santos JS, Lee DK, Ramamoorthy A. Effects of antidepressants on the conformation of phospholipid headgroups studied by solid-state NMR. Magn Reson Chem. 2004;42(2):105–14. 10.1002/mrc.1327 . 14745789

[15] Momo F, Fabris S, Stevanato R. Interaction of fluoxetine with phosphatidylcholine liposomes. Biophys Chem. 2005;118(1):15–21. 10.1016/j.bpc.2005.06.006 . 15994001

[16] Barry J, Fritz M, Brender JR, Smith PE, Lee DK, Ramamoorthy A. Determining the effects of lipophilic drugs on membrane structure by solid-state NMR spectroscopy: the case of the antioxidant curcumin. J Am Chem Soc. 2009;131(12):4490–8. 10.1021/ja809217u 19256547 PMC2748423

[17] Torrecillas A, Schneider M, Fernandez-Martinez AM, Ausili A, de Godos AM, Corbalan-Garcia S, et al Capsaicin Fluidifies the Membrane and Localizes Itself near the Lipid-Water Interface. ACS Chem Neurosci. 2015 10.1021/acschemneuro.5b00168 . 26247812

[18] Thennarasu S, Tan A, Penumatchu R, Shelburne CE, Heyl DL, Ramamoorthy A. Antimicrobial and membrane disrupting activities of a peptide derived from the human cathelicidin antimicrobial peptide LL37. Biophys J. 2010;98(2):248–57. 10.1016/j.bpj.2009.09.060 20338846 PMC2808482

[19] Porcelli F, Ramamoorthy A, Barany G, Veglia G. On the role of NMR spectroscopy for characterization of antimicrobial peptides. Methods Mol Biol. 2013;1063:159–80. 10.1007/978-1-62703-583-5_9 . 23975777 PMC4988059

[20] Hwang TC, Koeppe RE II, Andersen OS. Genistein can modulate channel function by a phosphorylation-independent mechanism: importance of hydrophobic mismatch and bilayer mechanics. Biochemistry. 2003;42(46):13646–58. 14622011 10.1021/bi034887y

[21] Lundbæk JA, Birn P, Tape SE, Toombes GE, Søgaard R, Koeppe RE II, et al Capsaicin regulates voltage-dependent sodium channels by altering lipid bilayer elasticity. Mol Pharmacol. 2005;68(3):680–9. 15967874 10.1124/mol.105.013573

[22] Bruno MJ, Koeppe RE II, Andersen OS. Docosahexaenoic acid alters bilayer elastic properties. Proc Natl Acad Sci USA. 2007;104(23):9638–43. 17535898 10.1073/pnas.0701015104 PMC1887599

[23] Ingólfsson HI, Koeppe RE II, Andersen OS. Curcumin is a modulator of bilayer material properties. Biochemistry. 2007;46(36):10384–91. 17705403 10.1021/bi701013n

[24] Rusinova R, Herold KF, Sanford RL, Greathouse DV, Hemmings HCJ, Andersen OS. Thiazolidinedione insulin sensitizers alter lipid bilayer properties and voltage-dependent sodium channel function: implications for drug discovery. J Gen Physiol. 2011;138(2):249–70. 10.1085/jgp.201010529 21788612 PMC3149818

[25] Ingólfsson HI, Thakur P, Herold KF, Hobart EA, Ramsey NB, Periole X, et al Phytochemicals perturb membranes and promiscuously alter protein function. ACS Chem Biol. 2014;9(8):1788–98. 10.1021/cb500086e 24901212 PMC4136704

[26] Huang HW. Deformation free energy of bilayer membrane and its effect on gramicidin channel lifetime. Biophys J. 1986;50(6):1061–70. 2432948 10.1016/S0006-3495(86)83550-0 PMC1329780

[27] Nielsen C, Andersen OS. Inclusion-induced bilayer deformations: effects of monolayer equilibrium curvature. Biophys J. 2000;79:2583–604. 11053132 10.1016/S0006-3495(00)76498-8 PMC1301140

[28] Lundbæk JA, Andersen OS. Cholesterol regulation of membrane protein function by changes in bilayer physical properties—an energetic perspective In: Levitan IB, Barrantes J, editor. Cholesterol Regulation of Ion Channels and Receptors. Hoboken, NJ: John Wiley and Sons, Inc.; 2012.

[29] Sawyer DB, Koeppe RE II, Andersen OS. Induction of conductance heterogeneity in gramicidin channels. Biochemistry. 1989;28(16):6571–83. 2477060 10.1021/bi00442a007

[30] Sawyer DB, Andersen OS. Platelet-activating factor is a general membrane perturbant. Biochim Biophys Acta. 1989;987(1):129–32. 2480815 10.1016/0005-2736(89)90464-1

[31] Lundbæk JA, Andersen OS. Lysophospholipids modulate channel function by altering the mechanical properties of lipid bilayers. J Gen Physiol. 1994;104:645–73. 7530766 10.1085/jgp.104.4.645 PMC2229230

[32] Lundbæk JA, Collingwood SA, Ingólfsson HI, Kapoor R, Andersen OS. Lipid bilayer regulation of membrane protein function: gramicidin channels as molecular force probes. J R Soc Interface. 2010;7:373–95. 10.1098/rsif.2009.0443 19940001 PMC2842803

[33] Zepik HH, Walde P, Kostoryz EL, Code J, Yourtee DM. Lipid vesicles as membrane models for toxicological assessment of xenobiotics. Crit Rev Toxicol. 2008;38(1):1–11. 10.1080/10408440701524519 . 18161501

[34] Søgaard R, Werge TM, Bertelsen C, Lundbye C, Madsen KL, Nielsen CH, et al GABA_A_ receptor function is regulated by lipid bilayer elasticity. Biochemistry. 2006;45(43):13118–29. 17059229 10.1021/bi060734+

[35] Herold KF, Sanford RL, Lee W, Schultz MF, Ingólfsson HI, Andersen OS, et al Volatile anesthetics inhibit sodium channels without altering bulk lipid bilayer properties. J Gen Physiol. 2014;144(6):545–60. 10.1085/jgp.201411172 25385786 PMC4242807

[36] Ingólfsson HI, Andersen OS. Screening for small molecules' bilayer-modifying potential using a gramicidin-based fluorescence assay. Assay Drug Dev Technol. 2010;8:427–36. 10.1089/adt.2009.0250 20233091 PMC2929145

[37] Darby CM, Ingólfsson HI, Jiang X, Shen C, Sun M, Zhao N, et al Whole cell screen for inhibitors of pH homeostasis in *Mycobacterium tuberculosi*s. PloS one. 2013;8(7):e68942 10.1371/journal.pone.0068942 23935911 PMC3728290

[38] Alejo JL, Blanchard SC, Andersen OS. Small-molecule photostabilizing agents are modifiers of lipid bilayer properties. Biophys J. 2013;104(11):2410–8. 10.1016/j.bpj.2013.04.039 23746513 PMC3672891

[39] Ingólfsson HI, Sanford RL, Kapoor R, Andersen OS. Gramicidin-based fluorescence assay for determining small molecules potential for modifying lipid bilayer properties. J Vis Exp. 2010;(44):e2131. 10.3791/2131 PMC3185632 20972414

[40] Berberan-Santos MN, Bodunov EN, Valeur B. Mathematical functions for the analysis of luminescence decays with underlying distributions 1. Kohlrausch decay function (stretched exponential). Chem Phys. 2005;315:171–82.

[41] Sanford RLC-G, J.; Luz A.; Glickman J. F.; Andersen O.S. Predicting drug toxicity: Early detection of likely failures in drug development. Biophys J. 2015;108.

[42] Seydel JK, Wiese M, editors. Drug-Membrane Interactions: Analysis, Drug Distribution, Modeling. Weinheim: Wiley-VCH; 2002.

[43] Bickerton GR, Paolini GV, Besnard J, Muresan S, Hopkins AL. Quantifying the chemical beauty of drugs. Nature Chem. 2012;4:90–8. 22270643 10.1038/nchem.1243 PMC3524573

[44] Viswanadhan VN, Ghose AK, Revankar GR, Robins RK. Atomic physicochemical parameters for three dimensional structure directed quantitative structure-activity relationships. 4. Additional parameters for hydrophobic and dispersive interactions and their application for an automated superposition of certain naturally occurring nucleoside antibiotics. J Chem Inf Comp Sci. 1989;29(3):163–72.

[45] Paul SM, Mytelka DS, Dunwiddie CT, Persinger CC, Munos BH, Lindborg SR, et al How to improve R&D productivity: the pharmaceutical industry's grand challenge. Nat Rev Drug Discov. 2010;9(3):203–14. 10.1038/nrd3078 . 20168317

[46] Bruno MJ, Rusinova R, Gleason NJ, Koeppe RE II, Andersen OS. Interactions of drugs and amphiphiles with membranes: Modulation of lipid bilayer elastic properties by changes in acyl chain unsaturation and protonation. Faraday Disc. 2013;161:461–80. 10.1039/c2fd20092a PMC3703894 23805753

[47] Lundbæk JA, Birn P, Hansen AJ, Søgaard R, Nielsen C, Girshman J, et al Regulation of sodium channel function by bilayer elasticity: the importance of hydrophobic coupling: effects of micelle-forming amphiphiles and cholesterol. J Gen Physiol. 2004;123:599–621. 15111647 10.1085/jgp.200308996 PMC2234500

[48] Fletcher S, Avery VM. A novel approach for the discovery of chemically diverse anti-malarial compounds targeting the Plasmodium falciparum Coenzyme A synthesis pathway. Malar J. 2014;13:343 10.1186/1475-2875-13-343 25174342 PMC4168161

[49] Hain AU, Bartee D, Sanders NG, Miller AS, Sullivan DJ, Levitskaya J, et al Identification of an Atg8-Atg3 protein-protein interaction inhibitor from the medicines for Malaria Venture Malaria Box active in blood and liver stage Plasmodium falciparum parasites. J Med Chem. 2014;57(11):4521–31. 10.1021/jm401675a 24786226 PMC4059259

[50] Lucantoni L, Duffy S, Adjalley SH, Fidock DA, Avery VM. Identification of MMV malaria box inhibitors of plasmodium falciparum early-stage gametocytes using a luciferase-based high-throughput assay. Antimicrob Agents Chemother. 2013;57(12):6050–62. 10.1128/AAC.00870-13 24060871 PMC3837862

[51] Paiardini A, Bamert RS, Kannan-Sivaraman K, Drinkwater N, Mistry SN, Scammells PJ, et al Screening the Medicines for Malaria Venture "Malaria Box" against the *Plasmodium falciparum* aminopeptidases, M1, M17 and M18. PloS one. 2015;10(2):e0115859 10.1371/journal.pone.0115859 25700165 PMC4336144

[52] Boyom FF, Fokou PV, Tchokouaha LR, Spangenberg T, Mfopa AN, Kouipou RM, et al Repurposing the open access malaria box to discover potent inhibitors of Toxoplasma gondii and Entamoeba histolytica. Antimicrob Agents Chemother. 2014;58(10):5848–54. 10.1128/AAC.02541-14 25049259 PMC4187973

[53] Alemán Resto Y, Fernández Robledo JA. Identification of MMV Malaria Box inhibitors of *Perkinsus marinus* using an ATP-based bioluminescence assay. PloS one. 2014;9(10):e111051 10.1371/journal.pone.0111051 25337810 PMC4206467

[54] Njuguna JT, von Koschitzky I, Gerhardt H, Lammerhofer M, Choucry A, Pink M, et al Target evaluation of deoxyhypusine synthase from *Theileria parva* the neglected animal parasite and its relationship to *Plasmodium* . Bioorg Med Chem. 2014;22(15):4338–46. 10.1016/j.bmc.2014.05.007 . 24909679

[55] Kaiser M, Maes L, Tadoori LP, Spangenberg T, Ioset JR. Repurposing of the open access Malaria Box for kinetoplastid diseases identifies novel active scaffolds against *Trypanosomatids* . J Biomol Screen. 2015;20(5):634–45. 10.1177/1087057115569155 . 25690568

