# Supplementary figures and images for: Bilayer Effects of Antimalarial Compounds
Source: PLoS One. 2015 Nov 9;10(11):e0142401. doi: 10.1371/journal.pone.0142401 (PMC4638347; doi:10.1371/journal.pone.0142401)

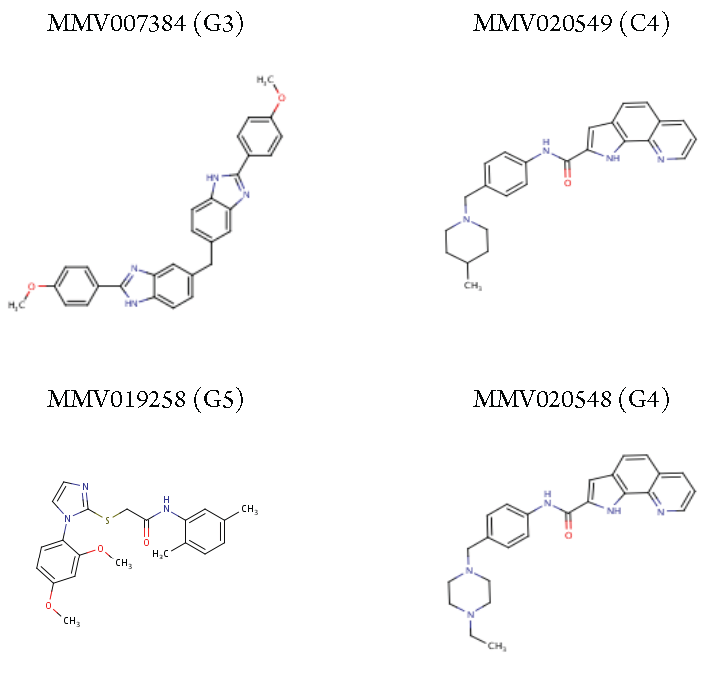

Supplement: S2 Fig — These four molecules, at 5 μM, increase the fluorescence quench rates statistically significantly in comparison to control (p<0.05; p<0.01 for MMV007384). (TIFF) [file pone.0142401.s002.tiff]
